# Evaluating the validity evidence of an OSCE: results from a new medical school

**DOI:** 10.1186/s12909-018-1421-x

**Published:** 2018-12-20

**Authors:** Vanda Yazbeck Karam, Yoon Soo Park, Ara Tekian, Nazih Youssef

**Affiliations:** 1 Lebanese American University-School of Medicine, P.O. Box: 113288, Zahar Street, Beirut, Lebanon; 2 Department of Medical Education, University of Illinois, Chicago, USA

**Keywords:** Objective structured clinical examination, Validity evidence, Quality assurance

## Abstract

**Background:**

To prevent the problems of traditional clinical evaluation, the “Objective Structured Clinical Examination (OSCE)” was presented by Harden as a more valid and reliable assessment instrument. However, an essential condition to guarantee a high-quality and effective OSCE is the assurance of evidence to support the validity of its scores. This study examines the psychometric properties of OSCE scores, with an emphasis on consequential and internal structure validity evidence.

**Methods:**

Fifty-three first year medical students took part in a summative OSCE at the Lebanese American University-School of Medicine. Evidence to support consequential validity was gathered by using criterion-based standard setting methods. Internal structure validity evidence was gathered by examining various psychometric measures both at the station level and across the complete OSCE.

**Results:**

Compared to our actual method of computing results, the introduction of standard setting resulted in lower students’ average grades and a higher cut score. Across stations, Cronbach’s alpha was moderately low.

**Conclusion:**

Gathering consequential and internal structure validity evidence by multiple metrics provides support for or against the quality of an OSCE. It is critical that this analysis be performed routinely on local iterations of given tests, and the results used to enhance the quality of assessment.

**Electronic supplementary material:**

The online version of this article (10.1186/s12909-018-1421-x) contains supplementary material, which is available to authorized users.

## Background

To prevent the problems of traditional clinical evaluation, the “Objective Structured Clinical Examination (OSCE)” was presented by Harden as a more valid and reliable assessment instrument [1]. However, an essential condition to guarantee a high-quality and effective OSCE is the assurance of evidence to support the validity of its scores [2].

The validity of a test is the degree to which this test measures what is intended to measure and hence the validity of a test should be accumulated by collecting several sources of evidence [3]. In 1989, Messick proposed a modern validity framework [4] that was considered a standard of practice in 1999 [5] and also in 2014 [6]. The theory behind Messick’s construct validity includes the evidence supporting the test development and the consequences of the results [4]. According to Messick’s framework, five sources of validity should be considered in order to accept or refute the scores generated by any assessment tool [4]. The five sources are: content (test items are characteristic of the construct of interest), response process (evidence of data coherence), internal structure (psychometric properties of the exam), relations with other variables (alignment of results with similar or different tools measuring the same subject) and consequences (impact on learners, instructors, and curriculum) [4].

The validity of the scores generated by any OSCE depends on its capability to appropriately sample the domain to be measured [7]. Ultimately, an effective OSCE should test cognitive, psychomotor, and affective skills. However, the OSCE is principally used for the assessment of the ‘*shows how*’ level of Miller’s pyramid [8]. Our purpose is to examine, using Messick’s theory as a conceptual framework, the construct validity of an OSCE we administered at the Lebanese American University – School of Medicine (LAU-SOM). We attempted to gather multiple sources of evidence with an emphasis on supporting internal structure and consequential validity. Unlike other validation studies, our investigation was based on a summative application of an OSCE where the validity of the score inferences is dependent, to a great extent, on the proper application of standard setting techniques. The other objective of the study was the use of the Borderline Regression Method (BRM) as a method for standard setting to determine the pass/fail cut scores and its comparison to our traditional method of computing the results.

## Methods

### Study participants

This study was conducted at the LAU-SOM, where a 4-year integrated curriculum is followed after a Bachelor’s degree. The assessment battery includes summative and formative tools, including OSCEs. The OSCE team at LAU comprises one drama teacher who recruits and trains standardized patients (SPs) and two physicians who write cases and develop checklists. Fifty-three first year medical students took part in a summative OSCE evaluating the hematology and endocrinology modules. Ethics approval was granted by the LAU Institutional Review Board. Using two simultaneous tracks and three consecutive testing periods, students were assessed on the same day. Each track included the same stations located in different rooms of the clinical simulation center. Each track comprised seven OSCE stations. Five stations consisted of patient encounters with an examiner and an SP or a manikin present in the room. The other two stations were pathology and microbiology and therefore were excluded from our analysis.

#### Content

Content evidence refers to ensuring that the construct being assessed is accurately and completely represented on a test [9]. The OSCE stations included various clinical skills related to the hematology and endocrinology modules: 1-ft exam, 2-neck exam, 3-couplet station: history taking patient with fatigue and write-up, 4-counseling for thalassemia, and 5-breast exam. Each station was 10 min except station three that lasted 30 min. Different content experts wrote and reviewed the cases that were pilot-tested prior to their implementation. Moreover, All checklists were developed in advance, following consultation with the content experts and in line with outcomes being assessed. The physician examiners (PEs) directly observed students’ performance and provided both grades; the checklist grades and the global rating grades. In addition, for stations assessing history taking and communication skills, a checklist scored by the SP was used and its grade added to the checklist grade with a weight of 10% (Additional file 1). At the end of the OSCE, the completed checklists were checked for their accurateness.

#### Response process

Response process ensures the correctness and the integrity of the data collected by the checklists to reduce any possible bias [6]. The validity of the final scores relates directly to the accuracy of the grades provided by the assessors. Physician examiners (PEs) were trained faculty from the School of Medicine. For this OSCE, they were provided with the appropriate instructions during a 2-h session in order to get familiar with the checklists’ items, the marking process, and the expected students’ behavior. Updating on the OSCE day reinforced the guidelines about the marking system. Checklists included 10–35 items for each station (Additional file 2). Each item was scored using a 3-point scale correlated to the task completion. The global rating score consisted of a 5-point scale associated with the overall performance of the student and based on the PEs’ global impression and not on the items’ scores. A hard copy of the global rating descriptors was kept in each station in the examiner file (Additional file 3). SPs were properly trained for their roles over three sessions, 2 h each. They were provided with the case details including their roles, any potential questions students may ask, and the appropriate answer for each question. During OSCE administration, the completeness of the checklist items and the global rating was monitored by dedicated staff after each round of students.

#### Consequences

**Fig. 1:**
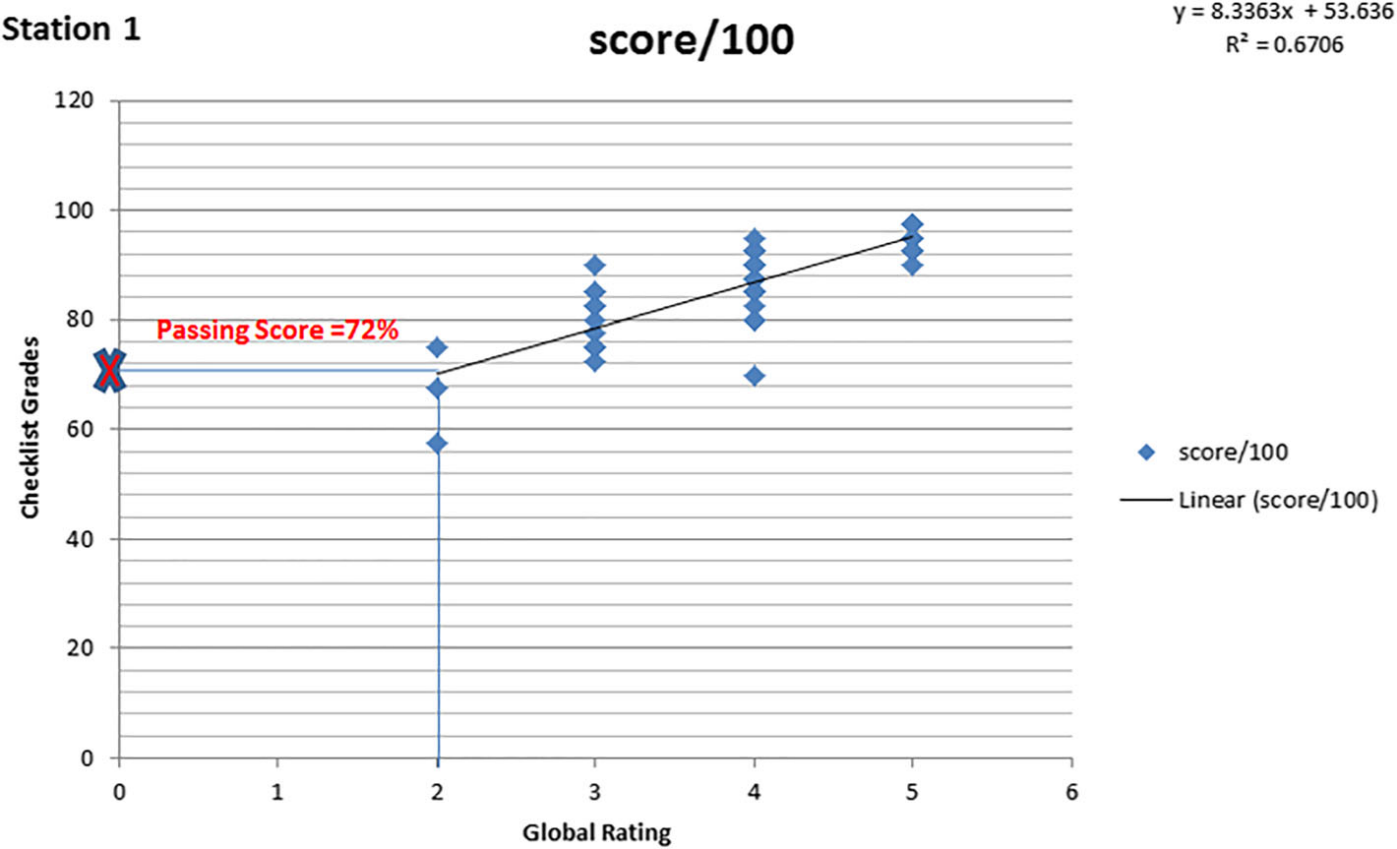
Example of calculating the BRM pass/fail standard with linear regression. Scatter plots of the checklist score versus the global rating score for station 1. The checklist score cut-off is calculated on the regression equation for the the global rating score scale cut-off set at 2. The passing score is 72

Consequential validity explores the real and latent impact of any test scores on examinees. Passing rates or cut-off scores are closely linked to the sources of consequential validity [10]. The passing score is the minimum score needed to demonstrate acceptable performance and pass the test. While standards may be set using random decisions, standard setting is a process that results in a credible and acceptable passing or cut-off scores in a logical and justifiable manner [11]. In our OSCE, the BRM was applied to establish a passing standard [12–14]. Checklists and global rating scores were reported separately for each station. We used the global rating solely for the calculation of standard setting. For each station, a linear regression model was utilized, with the consideration of the checklist as dependent variable and the global rating as independent variable. The BR pass/fail standard per station was obtained by using the regression line to calculate the checklist score corresponding with the cut-off point ‘2’ (borderline) of the global rating. An example for the calculation of the standard setting for station one is shown in Fig. 1. By inserting the point 2 of the global rating scale corresponding to the borderline group, a corresponding predicted checklist score could be determined. This predicted score 72 became the pass/fail standard for this station**.** The total test score was calculated by averaging the station checklist scores. The corresponding pass-fail standard for the five stations was defined as the average of the stations cut-scores, giving all stations a weight of one except station four with a weight of two since this is a couplet station that lasted 30 min. The pass/fail results of the OSCE using the borderline regression method (Method 2) were compared to our current method of computing the results (Method 1) that consists of ading the checklist grades with a weight of 75% to the global rating grades with a weight of 25%.

#### Internal structure

The internal structure validity evidence correlates to the psychometric measures of the test encompassing inter-item correlations, exam difficulty and score reliability. Reliability was evaluated using the following metrics: 1- Cronbach’s alpha measures the internal consistency whereby in an effective test, better students should perform similarly well in all stations. Acceptable alpha value in OSCEs where SPs are used is 0.7 or above. However, in lower stakes exam, a Cronbach’s alpha of a lesser value is acceptable; 2- *R*^2^ coefficient is the squared linear correlation between the holistic rating score and the checklist score. It is expected that the two scores be positively correlated. An *R*^2^ = 0.5 is considered reasonable; 3- inter-grade discrimination is the average increase in scores of the checklist for each grade increase on the holistic rating. An adequate discrimination index should be the tenth of the maximum score that could be generated by the checklist; 4- number of failures is used to review the quality of teaching and the need for change on a particular subject; 5- between group variation relates to the effect of the environment and assessor attitude on the scores rather than the performance of examinees. To note that in an ideal assessment process, all differences should be only due to student performance therefore between group variation should be under 30%, 6- standardized patient rating that is added to the checklist grade with a weight of 10% appears to be a robust way of incorporating such data, leading to the improved reliability of the assessment (Homer et al. 2009). If the SP rating is coupled with a higher than normal failure rate, this could be the result of inadequate teaching of the topic.

### Statistical analysis

Data were analyzed using Microsoft Excel, 2010. Characteristics of the study population were evaluated using descriptive statistics. Data were expressed as numbers and percentages for categorical variables and as means ± SD for continuous variables. Independent *t* tests were used for comparing means between the two groups. A value of *p* < 0.05 was considered significant. Rescaling was used to have the same passing score for both methods (60%) and to comply with the school policy for reporting.

## Results

Fifty-three students, 27 males and 26 females participated in this OSCE.

### Content

The OSCE blueprint represented five of the major objectives of the hematology-endocrinology module (Additional file 4). The scoring instruments included a station-specific analytical scoring or checklist developed by experts, a holistic score or five-point global rating scale, both filled by the PEs and a communication skills checklist filled by the SP.

### Response process

Our PEs and SPs underwent training sessions about the use of the different checklists. During the debriefing session following the OSCE, all reported being comfortable with its use.

### Consequences

The cut score for the 5 stations was: (72 × 1) + (60 × 1) + (53 × 1) + (70 × 2) + (67 × 1) = 65.16%. Using this cut score, the passing rate was 100%. Table 1 shows stations’ length, means and standard deviation, minimum and maximum grades, cut score as well as the percentage of pass rate and number of failures. Table 2 represents the compared results of the BRM (Method 2) to our actual method of computing the results (Method 1). Although the BRM method showed a lower students’ average grades (75.63 vs 79.23) and a higher cut score (65.16 vs 60), no statistical significance in scores between all stations was noted. However, when scores were rescaled to the cut score of 60%, as per our School policy, a statistical difference in the scores between the two methods for the overall grade and for all stations except for station 2 was noted. The passing rate was 100% for both methods because scores are aggregated across cases to provide a compensatory-type standard for the whole test.

**Table 1:** Stations name, length, means and standard deviation, minimum and maximum grades, cut score and percentage of pass rate and number of failures

Station Number	Station name	Station time (min)	Cut score %	Mean	Standard deviation	Min	Max	Number of failures	Pass rate %
1	Foot Exam	10	72	86.509	8.486	57.5	98	3	94.33
2	Neck Exam	10	60	85.031	12.928	46.7	100	1	98.11
3	Counseling Thalassemia	10	53	72.83	14.683	30	100	7	86.79
4	History Taking (Fatigue) + Write-up	30	70	76.528	8.632	58	96	10	81.13
5	Breast Exam	10	67	86.364	8.33	72.7	100	0	100
1–5		70	65.17	80.632	5.24	67	93	0	100

**Table 2:** The cut score, number of failures, pass rate, average grades and *p* value before and after rescaling for each method

	Method 1				Method 2					P value	
Station Number	Cut score %	Number of failures	Pass rate %	Average grade	Cut score %	Number of failures	Pass rate %	Average grade before rescaling	Average grade after rescaling	Before rescaling	After rescaling
1	60	1	98.11	84.981	72	3	94.33	80.73	74.509	0.4	5.60E-08
2	60	3	94.33	84.052	60	1	98.11	85.03	85.031	0.7	0.70337
3	60	8	84.9	72.151	53	7	86.79	77.36	80.83	0.8	0.00305
4	60	2	96.22	74.986	70	10	81.13	68.7	66.528	0.4	1.97E-06
5	60	0	100	84.277	67	0	100	83.47	79.364	0.2	0.00347
1–5	60	0	100	79.239	65.2	0	100	77.76	75.632	0.2	0.00108

### Internal structure

Across stations, Cronbach’s alpha in our OSCE was 0.43. The analysis of the different metrics showed an *R*^2^ value of 0.160 in station four, an inter-grade discrimination index of 13.55 in station three, the number of failures of 7 in station three (13.2%) and 10 in station four (18.86%). Between group variation was less than 30% and the number of failures was five (9.43%). The metrics of the different stations are shown in Table 3.

**Table 3:** Metrics of stations

Station Number	R^2^	Inter-grade discrimination	Number of failures	Between group variation %	Number of failures by SP ratings
1	0.670	8.33	3	86.509	0
2	0.669	12.67	1	85.031	0
3	0.598	13.55	7	6.021445	0
4	0.160	4.84	10	5.683655	5
5	0.568	11.5	0	1.962056	0

## Discussion

To establish the quality of an OSCE, evidence is needed to verify the validity of the scores. Moreover, one must also address possible threats to the validity of score-based inferences.

The consequential basis of validity implicates test grade analysis and use. Whereas the use of tests should consider the social consequences and their impact on trainees, teachers, and the whole curriculum, the interpretation of the tests’ results should consider the relationships between the favorable and unfavorable decisions that could be undertaken [4]. Choosing a defensible passing score by employing standard settings represents a persistent challenge to educators yet it is a key issue for ensuring the consequential basis of validity [15–17]. Nowadays, many institutions favor the borderline method that has several benefits [18]. First, it depends on the overall performance of trainees rather than the checklist markings and saves the clinicians’ time since the global rating is scored during the exam. Also, only three marks are required for global ratings (fail, borderline, pass) and the mean analytic scores of borderline students is the passing score of the exam, therefore it requires a simple statistical procedure. However, for the small-scale OSCE such as ours having a limited number of examinees, the presence of an only few examinees in the borderline range could introduce an unintentional bias. Pell et al. advised the use of the BRM that was initially described by Wood in 2005 [12, 19]. BRM is ideal in a small scale OSCE. It gives an indication of the relationship between global grade and checklist score by incorporating a linear regression approach allowing the cut score to be set using the scores from all examinees and not from a subset [14]. This method requires the use of five global ratings (e.g. fail, borderline, pass, very good pass, distinction) and more expertise for computation. However, it gives access to a wider variety of quality assurance metrics [13]. In our OSCE, the introduction of standard setting resulted in lower students’ average grades and a higher cut score.

The internal structure validity evidence involves the analysis of the different psychometric properties of the OSCE [20]. The reliability test scores can be evaluated using various indicators such as Generalizability, inter-rater reliability, rater consistency, and by the Coefficient alpha or Cronbach’s alpha, depending on the context of consistency evaluated [21]. Across stations, Cronbach’s alpha in our OSCE was 0.43 and is considered low. This could be explained by the low number of stations. Increasing the number of stations would result in greater reliability [7, 22]. This will have to be balanced against the feasibility in each setting. When a mismatch between the checklist and the global rating in a specific station is revealed, such in station four where the *R*^2^ value was low, this indicates that some students have acquired many of the marks from the analytic checklist for ‘process’, but their overall performance did not impress in parallel the examiner, suggesting that the checklists can be a poor marker of ability. Consequently, a redesign of the station should be made while focusing on matching criteria with the student level, inclusion of intermediate grade descriptors on the assessor checklists and ensuring that checklist criteria have three instead of two anchors where appropriate, thereby allowing greater discrimination by examiners. The presence of high failure rates at particular stations should lead to revisiting the teaching of a specific parts of the curriculum. In our OSCE, the high number of failures in station three and four highlighted teaching problems about counseling patients with thalassemia and conducting a history taking about fatigue and dizziness.

Threats to the validity of any assessment should be well-thought-out since the planning phase of an OSCE in order to avoid them. Two major threats to the validity are construct underrepresentation (CU) and construct-irrelevant variance (CIV) [23]. CU refers to the under sampling of the content domain by the use of insufficient number of cases, and to the inadequate sampling when the blueprint does not map the exam stations to the curriculum content and objectives. The blueprint of our OSCE included the content subdomains, the competencies to be assessed and patients’ characteristics. CIV is a systematic error introduced into the assessment data by variables unrelated to the construct being measured. CIV examples include flawed cases/checklists/rating scales, inappropriate difficulty level of the case, poorly trained standardized patients, or rater errors. The major CIV threat is due to systematic rater error. In fact, raters are a major source of measurement error, such as rater severity or leniency errors, central tendency error and halo rater effect. Therefore, upgrading training methods to improve between examiners’ agreement is essential in order to homogenize raters’ assessing skills. In addition, the provision of a detailed support material and briefings the examiners’ and SPs prior to the assessment should be systematically implemented. In this OSCE, content experts designed the checklists with carefully worded items and our examiners were trained faculty. Furthermore, the use of appropriate checklists/rating scales is critical [24]. Current evidence suggests that the use of holistic scoring or global rating scales by an experienced physician shows greater inter-station reliability, better construct validity, and better concurrent validity compared to checklists [25]. Global rating scales allow the examiner to rate the whole process compared to rating scales looking at one aspect alone specially when assessing areas such as judgment, empathy, organization of knowledge and technical skills [26, 27]. For OSCEs which use the BRM for establishing a standard setting, the use of the two types of checklists is mandatory.

Rigorous validation of educational assessments is critically important because those using an assessment must be able to trust the results [28]. Many schools use a predetermined cut scores for OSCE exams. However, setting defensible standards for student performance in an objective manner is critical, in particular when the OSCE is summative [29]. In this study, we have introduced a standard setting method and compared it to the preset cut score as per our school policy. We also analyzed the internal structure validity evidence by the use of multiple psychometric measures both at the individual station level and across the complete clinical assessment which allowed us to identify strengths and weaknesses of the quality of our OSCE scores.

A limitation to our study is the sample size of students as well as the number of OSCE stations. Another limitation is the generalizability of our results. We provided the evidence supporting the validity of a particular instantiation of an OSCE administered for one group of learners at our school. Understandably, larger sample sizes and wider school representation may have a varied impact on our results and warrants further investigation. However, our study is one of the few that was based on a summative application of an OSCE where the validity of the score inferences is largely dependent on the proper application of various quality assurance and standard setting techniques.

## Conclusion

OSCEs use criterion-based assessment principles within a complex process and constitute an integral part of the assessment system at many schools. The routine performance of a psychometric analysis on the OSCE results helps gaining an all-round view of the exam and prompts the identification and avoidance of common pitfalls.

Gathering consequential and internal structure validity evidence by multiple metrics provides support for or against the quality of an OSCE, in particular when used for a summative purpose. It is critical that this analysis be performed routinely on local iterations of given tests, and the results used to enhance the quality of assessment.

## Additional files

Additional file 1: Communication Skills Checklist filled by SPs. (DOCX 13 kb)

Additional file 2: History Taking Checklist. (DOCX 20 kb)

Additional file 3: Global Score Descriptors. (DOCX 14 kb)

Additional file 4: Blueprint of the OSCE for the Hematology-Endocrinology modules. (DOCX 15 kb)

## Abbreviations

BRM: Borderline regression method
CIV: Construct-irrelevant variance
CU: Construct underrepresentation
LAU-SOM: Lebanese American University-School of Medicine
OSCE: Objective Structures Clinical Examination
PEs: Physician Examiners
SPs: Standardized patients
