# Supplementary material for: Evaluating the validity evidence of an OSCE: results from a new medical school
Source: BMC Med Educ. 2018 Dec 20;18:313. doi: 10.1186/s12909-018-1421-x (PMC6302424; doi:10.1186/s12909-018-1421-x)
Supplement: Supplementary file 1 — Communication Skills Checklist filled by SPs. (DOCX 13 kb) [file 12909_2018_1421_MOESM1_ESM.docx]

Additional file 1: Communication Skills Checklist filled by SPs

1. The interviewer starts with open-ended questions (min2, max3) Yes □1 No □ 0

2. The interviewer has eyes contact during the interview Yes □1 Average □ 0.5 No □ 0

3. The interviewer verbally summarized at least once Yes □ 1 No □ 0

4. The interviewer non-verbal behavior is Good □ 1 Average □ 0.5 Poor □ 0

5. The interviewer shows empathy when patient expresses pain Good □ 1 Average □ 0.5 Poor □ 0

6. The interviewer showed a structured interview Good □1 Average □ 0.5 Poor □ 0

7. The interviewer asked more than one question at a time Yes □ 0 No □ 1

8. The interviewer asked leading questions Yes □ 0 No □ 1

**Overall Assessment:** Great □ 2 Good □1.5 Fair □1 Poor □ 0.5

**TOTAL = ……/10**

**Remarks:**
