# Supplementary material for: Evaluating the validity evidence of an OSCE: results from a new medical school
Source: BMC Med Educ. 2018 Dec 20;18:313. doi: 10.1186/s12909-018-1421-x (PMC6302424; doi:10.1186/s12909-018-1421-x)
Supplement: Supplementary file 2 — History Taking Checklist. (DOCX 20 kb) [file 12909_2018_1421_MOESM2_ESM.docx]

Additional 2: History Taking Checklist

**HISTORY CONTENT CHECKLIST**

| 0.5 | 0 |
| --- | --- |

1- Examiner introduces self and states role or position

| 0.5 | 0 |
| --- | --- |

2- Examiner asks or uses patient’s name

| 0.5 | 0 |
| --- | --- |

3- Examiner obtains verbal consent from the patient to take a history

**HISTORY OF PRESENT ILLNESS/CHIEF COMPLAIN**

| 1 | 0.5 | 0 |
| --- | --- | --- |

4- Onset of the symptoms

| 1 | 0.5 | 0 |
| --- | --- | --- |

5- Importance or severity of symptoms

| 1 | 0.5 | 0 |
| --- | --- | --- |

6- Previous episodes of fatigue/ dizziness

| 1 | 0.5 | 0 |
| --- | --- | --- |

7- Alleviating and exacerbating factors for fatigue

| 1 | 0.5 | 0 |
| --- | --- | --- |

8- Alleviating and exacerbating factors for dizziness

| 1 | 0.5 | 0 |
| --- | --- | --- |

9- Progression of the symptoms

| 1 | 0.5 | 0 |
| --- | --- | --- |

10- Syncope / gait problem

| 1 | 0.5 | 0 |
| --- | --- | --- |

11- Hearing problems/ visual problems

| 1 | 0.5 | 0 |
| --- | --- | --- |

12- Nausea/ Vomiting

| 1 | 0.5 | 0 |
| --- | --- | --- |

13- Headache

| 1 | 0.5 | 0 |
| --- | --- | --- |

14- Bowel habits? (Diarrhea, constipation)

| 1 | 0.5 | 0 |
| --- | --- | --- |

15- Shortness of breath

| 1 | 0.5 | 0 |
| --- | --- | --- |

16- Palpitations and chest pain

| 1 | 0.5 | 0 |
| --- | --- | --- |

17- Night sweats and sleeping disorders

| 1 | 0.5 | 0 |
| --- | --- | --- |

18- Skin changes? (Texture: dry, color: pale)

| 1 | 0.5 | 0 |
| --- | --- | --- |

19- Dark stools/ bloody urine

| 1 | 0.5 | 0 |
| --- | --- | --- |
|  |  |  |

20- Weight change/ decrease appetite

| 1 | 0.5 | 0 |
| --- | --- | --- |

21- Easy bleeding or bruising

| 1 | 0.5 | 0 |
| --- | --- | --- |

22- Heavy menses

| 1 | 0.5 | 0 |
| --- | --- | --- |

23- Medications (current and chronic)

| 1 | 0.5 | 0 |
| --- | --- | --- |

24- Personal history of anemia, transfusion?

| 1 | 0.5 | 0 |
| --- | --- | --- |
|  |  |  |

25- Family history of anemia

| 1 | 0.5 | 0 |
| --- | --- | --- |

26- Recent infection

**PAST MEDICAL HISTORY**

| 1 | 0.5 | 0 |
| --- | --- | --- |

27- PMH

| 1 | 0.5 | 0 |
| --- | --- | --- |

28- Surgeries

| 1 | 0.5 | 0 |
| --- | --- | --- |

29- Allergies

| 1 | 0.5 | 0 |
| --- | --- | --- |

30- Habits: smoking, alcohol, exercise

**FAMILY & SOCIAL HISTORY**

| 1 | 0.5 | 0 |
| --- | --- | --- |

31- Parents and family

| 1 | 0.5 | 0 |
| --- | --- | --- |

32- Examiner asks about social history

| 1 | 0.5 | 0 |
| --- | --- | --- |

33- Examiner asks about Stress / Mood

**REVIEW OF SYSTEMS**

| 1.5 | 0.5 | 0 |
| --- | --- | --- |

34-Examiner asks about other systems (eyes/ ears/ gastrointestinal/)

**Total Checklist = ……/ 33 + SP Checklist= ……./10**

**Global Rating: 1= Fail, 2= Borderline, 3= Pass, 4= Very good, 5= Outstanding**

**Remarks:**
