# Supplementary material for: Evaluating the validity evidence of an OSCE: results from a new medical school
Source: BMC Med Educ. 2018 Dec 20;18:313. doi: 10.1186/s12909-018-1421-x (PMC6302424; doi:10.1186/s12909-018-1421-x)
Supplement: Supplementary file 3 — Global Score Descriptors. (DOCX 14 kb) [file 12909_2018_1421_MOESM3_ESM.docx]

Additional file 3: Global Score Descriptors

| **5= Excellent** | Excellent performance of skill.  Outstanding demonstration of technical and non-technical aspects of skill.  Air of confidence and fluent. |
| --- | --- |
| **4= Very good pass** | Very good performance of skill.  Majority of the technical aspects of the skill demonstrated. Few minor and non-essential omissions / errors.  Examiner more than satisfied that candidate has passed station. |
| **3= Clear pass** | Acceptable performance of skill.  Despite omissions / errors demonstrated in performance of skill - safe to progress.  At times can be formulaic in approach. |
| **2= Borderline:**  ***pass doubtful*** | Patchy performance of skill. Examiner undecided whether to pass or fail candidate.  Demonstrated some aspects of the skill however omissions and inaccuracies occurred in their performance of the skill.  Often formulaic in approach and struggled with performing skill. |
| **1= Clear fail** | Performance of skill did not come up to a passing standard. Appeared disorganized.  Unsafe and unsuitable to progress. |
