# Supplementary material for: Evaluating the validity evidence of an OSCE: results from a new medical school
Source: BMC Med Educ. 2018 Dec 20;18:313. doi: 10.1186/s12909-018-1421-x (PMC6302424; doi:10.1186/s12909-018-1421-x)
Supplement: Supplementary file 4 — Blueprint of the OSCE for the Hematology-Endocrinology modules. (DOCX 15 kb) [file 12909_2018_1421_MOESM4_ESM.docx]

Additional file 4: Blueprint of the OSCE for the Hematology-Endocrinology modules

| **Station element** | **1** | **2** | **3** | **4** | **5** |
| --- | --- | --- | --- | --- | --- |
| **Content** | Diabetic Foot | Neck exam: Lymph nodes, thyroid, carotids | Thalassemia | Fatigue | Breast |
| **Task** | Examination of diabetic foot on SP | Examination of neck | Counseling about thalassemia Communication skills | History taking of a patient with fatigue  Write up and differential diagnosis | Examination of the breast |
| **Format** | Physical exam on SP | Physical exam  on SP | Counseling station with SP | History taking with SP + write up | Physical exam on manikin |
| **Length** | 10 minutes | 10 minutes | 30 minutes | 10 minutes | 10 minutes |
